# Differential responses of neurons, astrocytes, and microglia to G-quadruplex stabilization

**DOI:** 10.18632/aging.203222

**Published:** 2021-06-19

**Authors:** Natalie Tabor, Conelius Ngwa, Jeremie Mitteaux, Matthew D. Meyer, Jose F. Moruno-Manchon, Liang Zhu, Fudong Liu, David Monchaud, Louise D. McCullough, Andrey S. Tsvetkov

**Affiliations:** 1 Department of Neurology, The University of Texas McGovern Medical School at Houston, Houston, TX 77030, USA; 2 Institut de Chimie Moléculaire (ICMUB), UBFC Dijon, CNRS UMR6302, Dijon, France; 3 Shared Equipment Authority, Rice University, Houston, TX 77005, USA; 4 Biostatistics and Epidemiology Research Design Core Center for Clinical and Translational Sciences, The University of Texas McGovern Medical School at Houston, Houston, TX 77030, USA; 5 Department of Internal Medicine, The University of Texas McGovern Medical School at Houston, Houston, TX 77030, USA; 6 The University of Texas Graduate School of Biomedical Sciences, Houston, TX 77030, USA; 7 UTHealth Consortium on Aging, The University of Texas McGovern Medical School, Houston, TX 77030, USA

**Keywords:** G-quadruplex, DNA damage, genomic instability, neurodegeneration, Brca1

## Abstract

The G-quadruplex (G4-DNA or G4) is a secondary DNA structure formed by DNA sequences containing multiple runs of guanines. While it is now firmly established that stabilized G4s lead to enhanced genomic instability in cancer cells, whether and how G4s contribute to genomic instability in brain cells is still not clear. We previously showed that, in cultured primary neurons, small-molecule G4 stabilizers promote formation of DNA double-strand breaks (DSBs) and downregulate the *Brca1* gene. Here, we determined if G4-dependent *Brca1* downregulation is unique to neurons or if the effects in neurons also occur in astrocytes and microglia. We show that primary neurons, astrocytes and microglia basally exhibit different G4 landscapes. Stabilizing G4-DNA with the G4 ligand pyridostatin (PDS) differentially modifies chromatin structure in these cell types. Intriguingly, PDS promotes DNA DSBs in neurons, astrocytes and microglial cells, but fails to downregulate *Brca1* in astrocytes and microglia, indicating differences in DNA damage and repair pathways between brain cell types. Taken together, our findings suggest that stabilized G4-DNA contribute to genomic instability in the brain and may represent a novel senescence pathway in brain aging.

## INTRODUCTION

Guanine (G)-rich sequences in the human genome and transcriptome can fold into non-canonical structures known as G-quadruplexes (or G4s, G4-DNA and G4-RNA, respectively) [1, 2]. These sequences contain at least four G runs, which enable the four Gs to associate *via* Hoogsteen-type hydrogen-bonds to form self-stacking G-quartets, forming a columnar G4 structure, further stabilized by potassium cations in its inner channel [1]. Genomic G4s regulate transcription, replication, immunoglobulin gene recombination, and telomere function. More than 700,000 G4-DNA-forming sequences (QFS) were identified in the human genome by G4-seq [3]. QFS are often located near the replication start sites [4], as well as in oncogenes and regulatory genes and their promoters [5–7] and in mitochondrial DNA [8]. With G4 ChIP-seq analyses, however, the number of “active” G4-DNA structures was lower than predicted (*ca*. 10,000 QFS) and varied between cancerous and non-cancerous tissues and between cancer cell lines, demonstrating a cell type–specific G4-DNA landscape [9, 10]. G4-DNA-binding transcription factors, G4-DNA-associated proteins, and G4-DNA helicases bind to the G4-DNA structures and modulate G4 landscapes in cells [11–13]. In the human genome, variations in G4-DNA associated with single-nucleotide differences affect gene activity [14]. Nevertheless, the importance of G4s in cell physiology is also demonstrated by the fact that G4-DNA is often both evolutionary and phylogenetically conserved. In mammalian cells, G4-DNA structures are involved in recombination regions, including immunoglobulin class switch sites and at chromosomal translocation and deletion breakpoints [15, 16]. In yeast, G4-DNA structures promote DNA deletion, duplications, and gross chromosomal rearrangements [17, 18]. Importantly, numerous G4-associated proteins suppress genomic instability in yeast [12, 17, 19, 20], *Caenorhabditis elegans* [21], and cancer cells [22, 23]. For example, the G4 helicase PIF1 cooperates with breast cancer type 1 susceptibility protein (BRCA1) to unfold the G4-DNA structures at DNA double-strand breaks (DSBs) [24].

In addition to G4s, other alternative DNA structures exist in cells, such as triplex-DNA (or H-DNA), DNA junctions and DNA-RNA hybrids (R-loops). These highly thermodynamically stable structures act as physical roadblocks to the motion of polymerases along the genomic duplex, triggering DNA damage and leading to genomic instability [25]. Intriguingly, alternative DNA structures appear to regulate each other. For example, stabilized G4-DNA structures favor the formation of R-loops, which promote DNA damage and amplify genome instability in cancer cells [26]. Overall, alternative DNA structures represent an important endogenous source of genomic instability in cells [1].

Post-mitotic neurons must preserve their function throughout their lifespan. Transcriptionally active neurons need to cope with DNA damage and dedicate significant resources to maintain genome integrity and repair DNA damage [27]. Errors in repairing of DNA lesions that lead to non-reversible mutations, an age-associated decrease in DNA repair capacity, and age-dependent abnormal chromatin structure all lead to neuronal dysfunction and age-associated neurodegenerative disorders [27]. Dysfunctional DNA repair has been linked to many age-associated neurodegenerative diseases, including Alzheimer’s disease (AD), Parkinson’s disease, Huntington’s disease, and amyotrophic lateral sclerosis [28–30]. A failure of the nucleotide excision repair (NER), single-strand break repair (SSBR) or DSB repair pathways leads to neurological phenotypes [27]. Nucleotide bases in DNA are often modified by oxidation, alkylation, and deamination, resulting in DNA damage. For example, guanine oxidation is enhanced in the genes, which are downregulated in the aged human brain [31]. Oxidized Gs also readily form G4 structures [32], suggesting that they are important in aging.

In this study, we determined if G4-DNA-dependent DNA damage, chromatin structure, and *Brca1* downregulation are unique to neurons or if these effects are also relevant to astrocytes and microglia. We first showed that, basally, G4 landscapes differ among all three cell types. With electron microscopy, we found that pyridostatin (PDS), a selective G4-DNA-binding small molecule designed to form a stable complex with G4-DNA structures [33], induces significant chromatin re-arrangements in cultured cortical neurons, astrocytes, and to less extent microglia. We also showed that PDS induces DNA DSBs in primary cultured astrocytes and microglia, as well as in neurons. Intriguingly, unlike in neurons, PDS does not downregulate *Brca1* in astrocytes and microglial cells. With physico-chemical analyses, we confirmed that putative G4-forming sequences in the rat, mouse, human *Brca1* genes fold into the G4 structures. Our findings indicate that G4-DNA might be an important mechanism that induces genomic instability in brain cells in aging and neurodegeneration.

## RESULTS

### G4 landscapes differ among neurons, astrocytes and microglial cells

**Figure 1:**
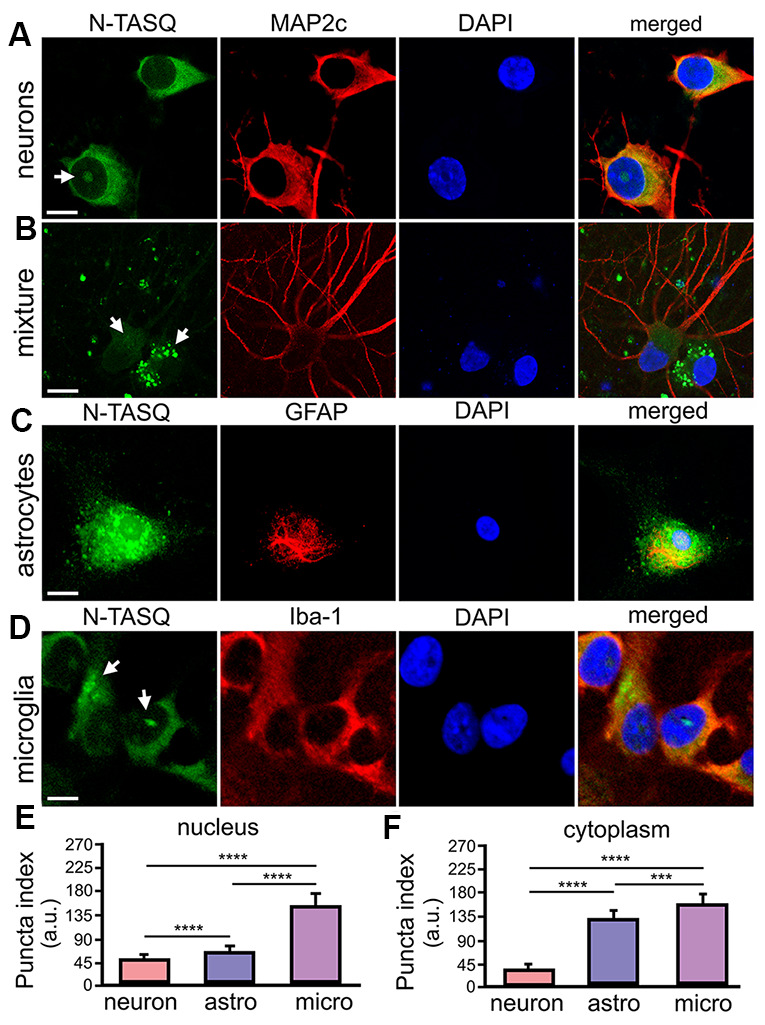
**G4 landscapes vary among primary neurons, astrocytes, and microglia.** (**A**) Primary cortical neurons (14 DIV) were fixed and stained with 25 μM N-TASQ, antibodies against MAP2c, and the nuclear dye DAPI, and imaged with a confocal microscope. Note the N-TASQ-positive structure in the nucleus of the cell on the left (depicted with arrow). Scale bar, 5 μm. (**B**) Primary cortical cultures were stained and imaged as in (**A**). Note the MAP2c-positive neurons on the right that contain small N-TASQ-positive puncta in the cytoplasm (depicted with arrow). Note the MAP2c-negative cell on the right that contains many N-TASQ-positive puncta in the cytoplasm (depicted with arrow). Scale bar, 10 μm. (**C**) Cultured primary astrocytes were fixed, stained with 25 μM N-TASQ, antibodies against GFAP, and DAPI, and imaged with a confocal microscope. Note numerous N-TASQ-positive structures in the nucleus and cytoplasm. Scale bar, 10 μm. (**D**) Cultured primary microglial cells were fixed, stained with 25 μM N-TASQ, antibodies against Iba-1, and DAPI, and imaged with a confocal microscope. Note N-TASQ-positive structures in the nuclei and the cytoplasm (depicted with arrows). Scale bar, 5 μm. (**E**) The nuclear puncta index of the N-TASQ staining was analyzed in the nuclei of cells from (**A**), (**C**, **D**). The Kruskal-Wallis test was used. ****p<0.0001. 120 cells per cell type were analyzed, and results were pooled from three independent experiments. (**F**) The cytoplasmic puncta index of the N-TASQ staining was analyzed in the cytoplasm of cells from (**A**), (**C**, **D**). The Kruskal-Wallis test was used. ****p<0.0001, ***p=0.0001. 120 cells per cell type were analyzed, and results were pooled from three independent experiments.

DNA damage and repair mechanisms could be, at least partially, attributed to a unique G4 landscape(s) that basally exists in a particular cell type. For example, G4 landscapes may vary in post-mitotic neurons and dividing glial cells. The G4 fluorophore NaphthoTASQ (N-TASQ), a “twice-as-smart ligand” that is a G4 ligand and fluorescent probe simultaneously [34], has been used to investigate G4 landscapes in fixed cells [34–38]. We, therefore, sought to compare if and how G4 landscapes differ among these major brain cell types. Cultured primary cortical neurons were fixed and stained with N-TASQ to assess a basal G4 landscape in neurons. Confocal microscopy revealed that N-TASQ staining is mostly diffuse in the neuronal cytoplasm or exhibits small puncta (Figure 1A, 1E, 1F). In some neuronal nuclei, however, large N-TASQ-positive *foci* or clusters of TASQ-positive *foci* reflect G4-enriched structures.

While analyzing neuronal cultures, which also contain a small amount of residual glial cell contamination, we noticed that there is a dramatic difference in pan N-TASQ staining in neurons and their symbiotic glial cells (Figure 1B). Although some neurons exhibit cytoplasmic TASQ-positive *foci*, the cytoplasm of glial cells is highly packed with N-TASQ-positive puncta (Figure 1B). Primary astrocytes and microglia were then cultured in parallel, stained with N-TASQ, and analyzed. We discovered that astrocytes contain high levels of N-TASQ *foci* in the cytoplasm and in the nucleus (Figure 1C, 1E, 1F). A similar pattern was also observed in cancerous cells, in which cytoplasmic N-TASQ *foci* mostly consisted of ribosomal RNA and long non-coding RNA, which fold into the G4-RNA structures [39]. Primary microglial cells vary considerably in their G4 landscapes (Figure 1D, 1E, 1F). Microglial cells often contain a large N-TASQ-positive structure or two in the nucleus, and many microglial cells exhibit cytoplasmic N-TASQ puncta—G4-RNA—similarly to astrocytes. We, therefore, conclude that, basally, G4-landscapes differ among neurons, astrocytes, and microglia, and there is some variation within these cell types.

### PDS differentially alters chromatin structure in primary neurons, astrocytes and microglia

Here, we determined if pharmacologically stabilizing G4-DNA promotes a re-arrangement of chromatin in primary cortical neurons, astrocytes, and microglia. Cell cultures were treated with a vehicle or PDS and imaged with transmission electron microscopy (TEM).

**Figure 2:**
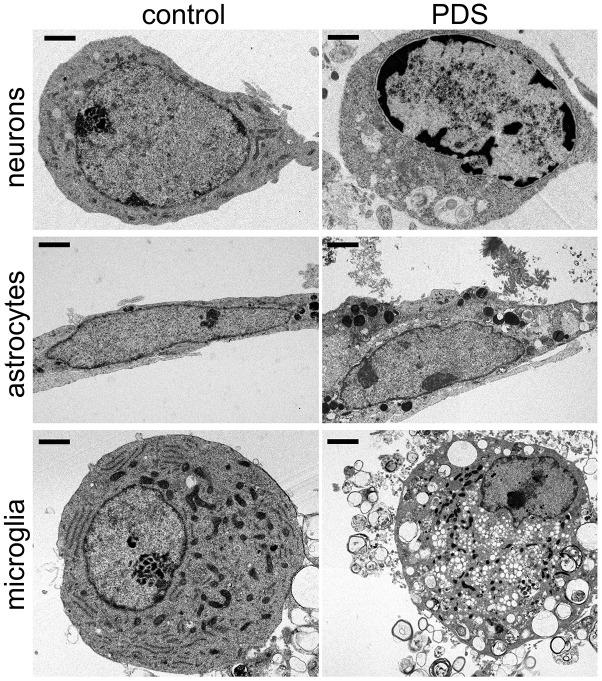
**PDS alters the structure of chromatin and cytoplasm in primary neurons, astrocytes, and microglia.** Representative electron micrographs of cultured cortical neurons, astrocytes, and microglia treated with a vehicle overnight (left panel) and with 2 μM PDS overnight (right panel). Bar (neuronal cells), 1 μm; bar (astrocytes and microglia), 2 μm. Primary cultures were fixed and processed for electron microscopy imaging. Results were pooled from two independent experiments.

We discovered that control vehicle-treated cultures had healthy neurons with normally organized cytoplasmic ultrastructural features, such mitochondria (Figure 2). Nuclei in vehicle-treated neurons generally exhibited a normal phenotype, with more heterochromatin localized near the nuclear membrane and a typical distribution and morphology of both euchromatin (electron-lucent) and heterochromatin (electron-dense) (Figure 2). Neurons treated with PDS exhibited more cytoplasmic variation by having uncharacteristic ultrastructural features, such as abnormal vesicles (Figure 2). Nuclei in PDS-treated neurons generally exhibited disorganized and highly variable chromatin morphology, including euchromatin and heterochromatin. Similar chromatin disorganization and electron-dense structures are observed in age-associated neurodegenerative diseases [40] and aged neurons [41, 42]. Additionally, abnormally shaped nuclei were common in PDS-treated neurons. Therefore, we conclude that PDS induces multiple abnormalities in cultured primary neurons, including aberrantly structured chromatin.

Primary astrocytes were cultured from embryonic rats and treated with PDS overnight. Control astrocytes appear to be of the lamellar subtype, and their long cell bodies contained numerous dense bodies [43]. Nuclei contained heterochromatin localized near the nuclear membrane (Figure 2). Nucleoplasms contained evenly distributed heterochromatin and euchromatin and one nucleolus per nuclei (Figure 2). PDS-treated astrocytes also contained numerous lamellar dense bodies, but cell bodies were more irregularly shaped and did not have the characteristically long cell body morphology (Figure 2). Cytoplasms of PDS-treated astrocytes often contained abnormal vesicles, such as autophagosome-like structures, that were also observed in neurons. In contrast to control vehicle-treated astrocytes, many nuclei in PDS-treated cells contained two nucleoli (Figure 2). However, the effects of PDS on astrocytic chromatins were considerably less dramatic.

Next, primary microglial cells were cultured from embryonic rats and treated with PDS overnight (Figure 2). Intriguingly, we found no major differences in ultrastructural chromatin morphology in control and PDS-treated microglia. The nuclei of both groups had nuclei with variable chromatin morphology. Nucleoli were also variable in control and PDS-treated cells (Figure 2). Nevertheless, PDS-treated microglial cells contained more autophagosomes, as PDS downregulates the autophagic flux [37, 38]. Our data indicate that neurons and glial cells respond differently to the G4-stablizing drug PDS, with neurons being more sensitive to PDS.

### PDS induces DNA damage in primary neurons, astrocytes and microglial cells

**Figure 3:**
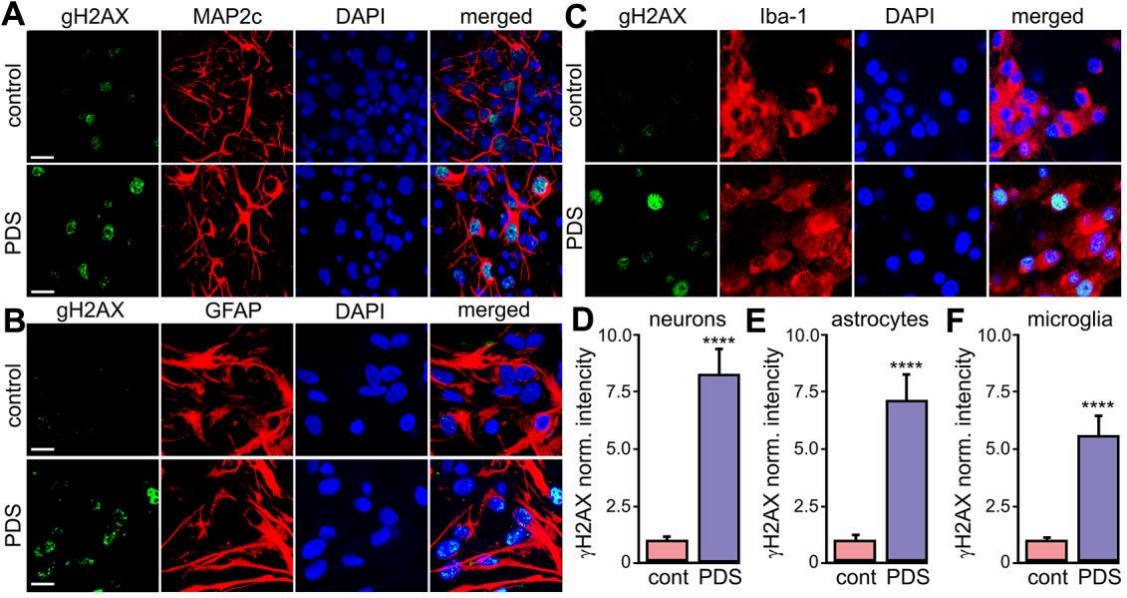
**PDS induces DNA DSBs in primary neurons, astrocytes, and microglia.** (**A**) Primary cortical neurons were treated with a vehicle (upper panel; control) or with 2 μM PDS (lower panel; PDS) overnight, fixed, and stained for a marker of DNA DSBs phosphorylated histone H2A variant X, γH2A.X (green), MAP2c (red), and with the nuclear DAPI dye (blue). Samples were imaged with a confocal microscope. Scale bar is 10 μm. (**B**) Primary astrocytes were treated with a vehicle (upper panel; control) or with 2 μM PDS (lower panel; PDS) overnight, fixed, and stained for γH2A.X (green), GFAP (red), and with DAPI (blue). Samples were imaged with a confocal microscope. Scale bar is 10 μm. (**C**) Primary microglial cells were treated with a vehicle (upper panel; control) or with 2 μM PDS (lower panel; PDS) overnight, fixed, and stained for γH2A.X (green), Iba-1 (red), and with the nuclear DAPI dye (blue). Samples were imaged with a confocal microscope. Scale bar is 10 μm. (**D**) The γH2A.X intensities were measured in images from (**A**) and normalized (arbitrary units). ****p<0.0001 (t-test). 100 neurons were analyzed from three independent experiments. (**E**) The γH2A.X intensities were measured in images from (**B**) and normalized (arbitrary units). ****p<0.0001 (t-test). 100 astrocytes were analyzed from three independent experiments. (**F**) The γH2A.X intensities were measured in images from (**C**) and normalized (arbitrary units). ****p<0.0001 (t-test). One hundred microglial cells were analyzed from three independent experiments.

We found that pharmacologically stabilizing G4-DNA promotes the formation DNA DSBs in primary cortical neurons [44]. Cancerous cells exhibit DNA damage upon exposure to G4-DNA ligands [45, 46]. Nevertheless, glial cells may respond differently to small-molecule G4-DNA stabilizers [47]. Primary astrocytes and microglia were cultured and treated with a vehicle or PDS overnight, fixed and stained for γH2A.X. Neurons, astrocytes and microglial cells exposed to PDS had γH2A.X punctate staining in the nuclei (Figure 3A–3F). Our data indicate that stabilizing G4s with PDS promotes DNA damage in neurons, as well in primary astrocytes and microglia.

### PDS downregulates *Brca1* in neurons, but not in astrocytes and microglial cells

**Figure 4:**
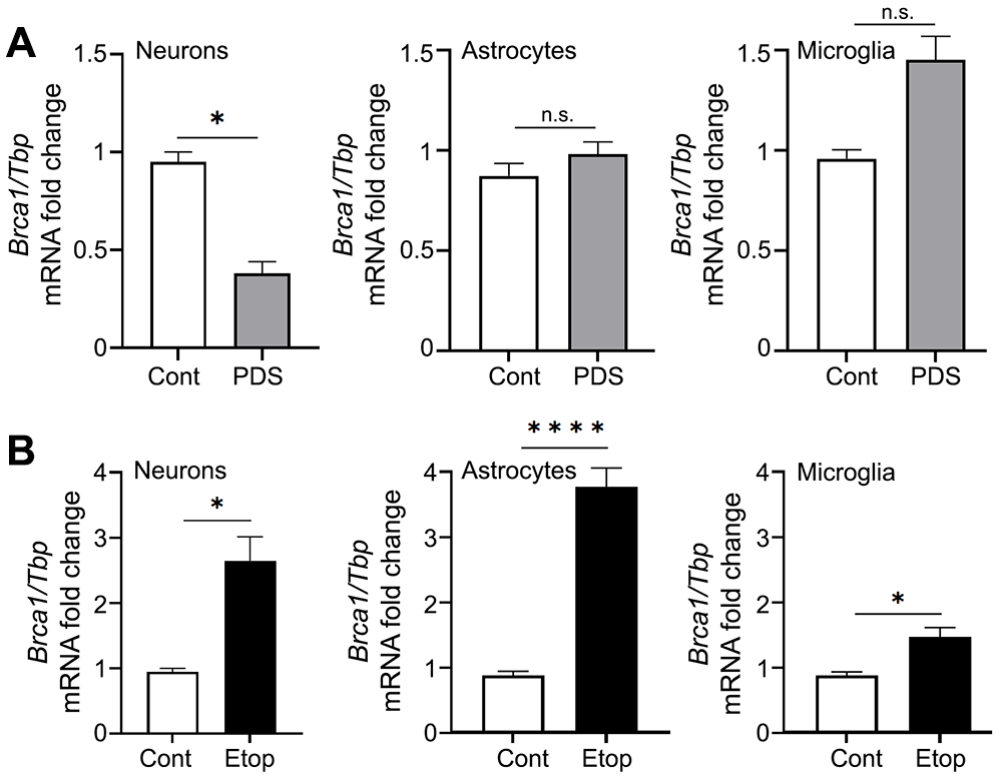
**PDS downregulates *Brca1* in primary neurons but not in primary astrocytes and microglial cells.** (**A**) Primary cortical neurons, astrocytes and microglial cells were treated with a vehicle or with 2 μM PDS overnight (18 h). Neurons were collected and lysed, and *Brca1* expression was determined by qRT-PCR normalized to *Tbp.* *p=0.05 (t=4.304> t_0.975,2_ = 4.303; significant). Results were pooled from duplicates of 2–4 independent reactions. (**B**) Primary cortical neurons, astrocytes and microglial cells were treated with a vehicle or with 2 μM etoposide overnight (18 h). Cells were collected and lysed, and *Brca1* expression was determined by qRT-PCR normalized to Tbp. *p<0.038, ****p< 0.0001 (t-test). Results were pooled from duplicates of 2–4 independent reactions.

We then wondered if G4-DNA-dependent *Brca1* downregulation is specific for neurons or if it also occurs in astrocytes and microglia. Primary microglia and astrocytes were treated with a vehicle or PDS, and mRNAs were extracted and analyzed. Since the *Tbp* gene does not contain PQFS and *Tbp* transcription is not affected by PDS [44], TBP mRNA was used as loading control. PDS did not downregulate *Brca1* in astrocytes and microglia, but it did reduce levels of *Brca1* mRNA in primary cortical neurons (Figure 4A). Our data indicate that *Brca1* in neurons is specifically sensitive to PDS, suggesting a cell-type-specific G4-DNA landscape in these cells.

PDS may alter DNA topology by trapping topoisomerase II on DNA [46]. To exclude the topoisomerase II component in effects we observed with PDS, we used an inhibitor of topoisomerase II, etoposide. We previously showed that etoposide promotes DNA damage in neurons [48]. Primary cortical neurons, astrocytes and microglia were treated with a vehicle or etoposide, and *Brca1* and *Tbp* mRNA were extracted and analyzed. In the three cell types tested, etoposide upregulated the *Brca1* gene (Figure 4B). Our data indicate that PDS and etoposide treatments modulate different DNA-associated molecular mechanisms in primary neurons, astrocytes, and microglia.

### The *Brca1* gene and its promoter contain multiple putative G4-DNA-forming sequences

We previously found that, in the rat *Brca1* gene promoter, only one of the computationally predicted two QFSs is recognized by the G4-DNA-specific antibody BG4 [44], suggesting a complex nature of G4-DNA in *Brca1* and its promoter. We used the QFS mapper to describe Brca1 and its promoter in several species. In *Homo sapiens, Brca1* has two putative G4-DNA sequences, and its promoter has one QFS. In *Mus musculus*, *Brca1* has five putative G4-DNA sequences, and its promoter has one PQFS. In *Rattus norvegicus, Brca1* has six putative G4-DNA-forming sequences, and its promoter has two G4-DNA-forming sequences [44] (Supplementary Figure 1).

Using a different algorithm to predict G4-DNA in both the sense and antisense strands, the G4Hunter software revealed more putative G4-DNA-forming sequences in *Brca1* (with a GH score ≥ 1.5). In *H. sapiens*, the gene contains 22 putative G4-DNA sequences and its promoter four. In *M. musculus*, the gene contains 23 putative G4-DNA sequences and its promoter four. In *R. norvegicus, Brca1* contains 25 putative G4-DNA-forming sequences and its promoter five (Supplementary Figure 1). Therefore, we conclude that there could be a higher degree of entanglement of *Brca1* regulation. *Brca1*’s G4-DNA may behave differently during *Brca1* transcription and replication, which may result in differences in post-mitotic and mitotic cells.

### Putative G4 sequences from *Brca1* and its promoter fold into G4 structures *in vitro*

**Figure 5:**
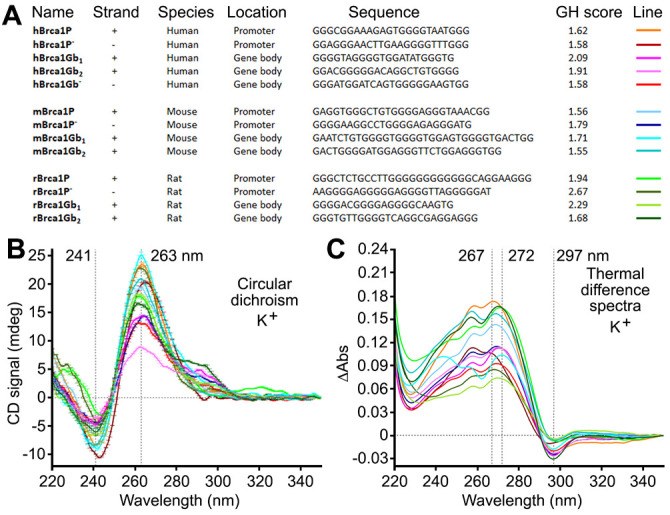
**Putative G4-forming sequences from the human, mouse, and rat *Brca1* and their promoters fold into G4-DNA structures *in vitro*.** (**A**) Sequences of putative G4-DNA-forming sequences from the human, mouse, and rat *Brca1* and their promoters, along with their G4Hunter (GH) scores. (**B**, **C**) CD and TDS signatures of these G4-forming sequences (3 μM) in Caco.K10 buffer (10 mM lithium cacodylate buffer (pH 7.2) plus 10 mM KCl and 90 mM LiCl).

The G4-forming sequences located near the transcription start site influence the expression of a gene most [7]. Using a series of established physico-chemical analyses (circular dichroism and thermal difference spectra (TDS)) [49], we confirmed that all identified sequences (in the human, mouse and rat genomes) fold into the G4 structures (Figure 5 and Supplementary Figures 2–10). Experiments in Na^+^- and K^+^-containing buffers provided CD/TDS signatures indicative of a stable G4 structure almost exclusively of a parallel-type in K^+^-conditions (*i.e.*, major contributions at 241 (negative) and 263 nm (positive) in CD, at 267–272 (positive) and 297 nm (negative) in TDS) (Figure 5), and less stable and more polymorphic in Na^+^-conditions (Supplementary Figure 2), as expected. Additionally, CD-melting experiments in the presence of PDS indicated that PDS stabilizes all these G4 structures, although to a different extent (Supplementary Figures 8–10). We, therefore, conclude that putative G4-forming sequences near the transcription initiation site in *Brca1* in the human, mouse, and rat genomes fold into G4s *in vitro*, likely regulating transcription and replication of the *Brca1* gene *in vivo*.

## DISCUSSION

Our findings establish a potentially novel mechanism of genomic instability in major brain cell types. PDS promotes DNA DSBs in cultured primary neurons, astrocytes and microglial cells but, intriguingly, the drug does not downregulate the levels of *Brca1*’s mRNA in astrocytes and microglia, as it does in neurons. Our data indicate that, in general, G4-DNA-stabilizing molecules promote DNA damage in neurons, astrocytes, and microglia *in vivo*, contributing to genomic instability in the brain. Thus, cell-type-specific differences in G4 landscapes, in health and disease, associated with DNA damage and repair pathways may contribute to cellular susceptibility to cell senescence.

Genomic stability is paramount for central nervous system (CNS) function and requires an intricate guarding and repair system to protect genome integrity. DNA replication is an important source of genomic instability during cell division, especially during the period of CNS development. High levels of oxidative metabolism, topoisomerase activity, and transcription result in DNA damage in the developed CNS. In the aging CNS, deficits in chromatin remodeling and abnormal chromatin re-arrangements lead to the impaired accessibility of repair factors to DNA. Cell-type-specific mechanisms of DNA damage and repair add more complexity to the preservation of genome integrity in the developing, mature, and aging CNS [47]. Our data demonstrate that cell responses to a G4 ligand, PDS, differ among neurons, astrocytes, and microglia. Future studies will improve our understanding of the cell-type-specific mechanisms involved in G4-DNA pathways.

Many proteins, including the G4-DNA processing helicases, that regulate G4-DNA are linked to human diseases [50, 51]. In Fanconi anemia, the FANCJ G4 resolving helicase is mutated, leading to genomic instability, bone marrow failure, and cancer [51]. Warsaw breakage syndrome, with less than a dozen patients identified, is caused by a mutation in the G4-DNA helicase DDX11 [51]. A severe multisystem bone-marrow-failure syndrome, dyskeratosis congenita, is linked to mutations in RTEL1, a helicase that processes telomeric G4-DNA [51]. Mutations in the helicase XPD lead to xeroderma pigmentosum, Cockayne syndrome, and other rare diseases [51]. Helicase WRN is mutated in Werner syndrome, which is characterized by genomic instability, accelerated aging, cardiovascular disease, and cancer [51]. Mutated helicase BLM causes Bloom syndrome, which is also associated with genomic instability and cancer [51]. The L319P mutation in the helicase PIF1 is associated with cancer [52]. Mutations in the telomere maintenance complex (the CST complex; CTC1, STN1, and TEN1) lead to cerebroretinal microangiopathy (Coats plus syndrome) with a failure of multiple organs [53]. All these diseases, perhaps excluding PIF1^L319P^-linked breast cancer, are characterized by some degree of brain pathology, often severe, that is likely linked to genomic instability. Our data indicate that cell-type-specific mechanisms of G4-DNA helicase functions may be involved.

In neuronal cells, the PDS/DNA complex likely stalls DNA polymerase during transcription to downregulate *Brca1* expression. Endonucleases may damage DNA by poisoning repair coupled to transcription [54]. Stabilization of R-loops, a nucleic acid structure consisting of two antiparallel DNA strands and an RNA strand, also results in DNA DSBs in PDS-treated neurons as it does so in non-neuronal cells [26, 55]. *Brca1* downregulation may further impede DNA damage repair in neurons. Critically, DNA DSBs are more perilous for post-mitotic neurons than dividing cells, which effectively repair DSBs by homologous recombination in sister chromatids [47]. To repair DNA DSBs, neurons should rely on a non-homologous end-joining mechanism that depends on error-prone DNA polymerases [56, 57]. Intriguingly, we found that, in primary astrocytes and microglial cell line, PDS promotes DNA DSB formation without downregulating *Brca1*, suggesting a different mechanism of PDS-associated DNA damage in glial cells. In dividing brain cells, PDS may promote DNA damage via a replication-dependent mechanism, as in cancer cells [45]. Because *Brca1* is not downregulated in PDS-treated astrocytes and microglial cells, mitotic cells may have specific G4-DNA resolving mechanisms during transcription. In addition, G4-DNA may function differently during *Brca1* transcription and replication in Braca1 and its promoter. In addition, G4 regulation may differ between species [58].

Is G4-DNA formation always pathogenic? Indeed, G4-DNA-associated DNA damage and genomic instability, DNA polymerase stalling, transcription repression, replication stalling, and nucleosome eviction are associated with cytotoxicity or carcinogenesis [59, 60]. Paradoxically, G4-DNA may upregulate the expression of certain genes by facilitating transcription factor binding to these genes and their promoters or by enabling re-initiation of transcription [13]. Telomeric DNA consists of G-containing repeats with the G-enriched strand being longer than its complement, resulting in formation of G4-DNA with telomeric G4-DNA-binding proteins bound to it [1]. Remarkably, genome-wide analysis of replication origins revealed that replication initiation sites are enriched in G4-DNA motifs, indicating that G4-DNA likely functions in replication by recruiting replication-activating proteins [61]. In addition to structuring the telomeres, G4-DNA appears to function in overall three-dimensional chromatin organization and in enhancer–promoter interaction [62]. Therefore, G4-DNA has beneficial roles, and future studies will define these beneficial mechanisms in neurons and glial cells.

In summary, we found that G4 landscapes differ among major brain cells—neurons, astrocytes, and microglial cells. A small-molecule ligand diminishes *Brca1* expression in neurons, but not in astrocytes and microglial cells, suggesting different mechanisms in glial cells. Our data suggest that G4-DNA contributes to genomic instability in brain cells, leading to brain aging and neurodegeneration.

## MATERIALS AND METHODS

### Care of rats

Rats were maintained in accordance with guidelines and regulations of the University of Texas McGovern Medical School at Houston (the protocol number #AWC-16-0081). All experimental protocols were approved by the University of Texas McGovern Medical School at Houston. The methods were carried out in accordance with the approved guidelines.

### Chemicals and antibodies

PDS was from Cayman Chemical (18013) and Selleck Chemicals (S7444). Hoechst dye was from Santa Cruz Biotechnology (sc-394039). Etoposide was from Selleck Chemicals (S1225). N-TASQ was synthesized as described [34]*.* Mouse antibodies against MAP2c were from Santa Cruz Biotechnology (1:100; sc-74421). Mouse antibodies against Iba-1 were from Santa Cruz Biotechnology (1:100; sc-32725). Rabbit antibodies against *γH2A.X* were from Abcam (1:5000; ab11174). Anti-mouse Alexa Fluor 546-labeled (#A-11003) and anti-rabbit Alexa Fluor 488-labeled (#A-11008) secondary antibodies were from Thermo Fisher Scientific.

### Cell cultures

Cortices from rat embryos (E17–18) were dissected, dissociated, and plated on 12-well tissue-culture plates (4x10^5^/well) for neurons and t-75 flasks for glial cells. All plates/flasks were coated with poly-D-lysine (Sigma-Aldrich, P6407), as described [37, 38, 63]. Primary cortical neurons were grown in Neurobasal Plus Medium (Gibco, 21103049), supplemented with B-27 (Gibco, 17504001) and penicillin-streptomycin (Gibco,15140122). Microglial cells were purified by shaking mixed glial cultures, collecting detached microglial cells and seeding them as a mono-culture in a 12-well plate. Microglial cells were grown in Dulbecco′s Modified Eagle Medium (Gibco, 11965118), supplemented with 10% heat-inactivated fetal bovine serum (Cytiva, SH30396.03HI), penicillin-streptomycin (Gibco, 15140122), and L929 conditioned medium. Remaining primary cortical astrocytes were grown in Dulbecco′s Modified Eagle Medium (Gibco, 11965118), supplemented with 10% heat-inactivated fetal bovine serum (Cytiva, SH30396.03HI) and penicillin-streptomycin (Gibco, 15140122) for at least 3 weeks.

### L929 conditioned media

L929 cells were cultured to full confluence on 75-cm^2^ flasks (Corning) in Dulbecco′s Modified Eagle Medium (HyClone, SH3024301) supplemented with 10% heat-inactivated fetal bovine serum (Sigma, F4135) and penicillin-streptomycin (Thermo Fisher Scientific, 15070063). Medium was aspirated, and cells were rinsed in mMG Medium and then cultured in 35 mL of mMG Medium. After 2 weeks, L929 conditioned medium was collected, filtered, and frozen at -80° C.

### Transmission electron microscopy

Primary cortical neurons were cultured for 2 weeks. The first neuronal cohort was treated with a vehicle overnight. The second cohort was treated with 2 μM PDS overnight. Neurons were fixed overnight in Karnovsky’s fixative, post-fixed in 1% osmium tetroxide, dehydrated using a graded series of ethanol and acetone, embedded in epoxy resin and heat-polymerized. Ultra-thin sections were cut at 100 nm on a Leica EM UC7 ultramicrotome (Leica, Buffalo Grove, IL) and stained with saturated methanolic uranyl acetate and lead citrate. Sections were examined using a JEOL JEM-1230 TEM (JEOL, Peabody, MA, USA), equipped with a Gatan Ultrascan (Gatan, Pleasanton, CA, USA) digital camera.

### G4-DNA analyses

The QGRS mapper (http://bioinformatics.ramapo.edu/QGRS/analyze.php) and the G4 Hunter (https://bioinformatics.cruk.cam.ac.uk/G4Hunter/) were used to determine the potential G4-DNA structures contained in genes of interest. Search parameters for the QGRS mapper were maximal length: 45; minimal G-group size: 3; loop size: from 0 to 10 [64, 65]. Search parameters for the G4 Hunter were threshold: 1.5; window size: 25 [66].

### RNA extraction and qRT-PCR

Total RNA was extracted from primary cultures with the RNeasy Mini kit (#74104, Qiagen). The following adjustments were made: 300 μL of Qiazol, 70 μL of chloroform, 225 μL of ethanol, 300 μL of buffer RWI, and 215 μL of buffer RPE. RNA was quantified using Nanodrop to determine how many μL of RNA to add to the cDNA reaction at 400 micrograms, and the RNA was reverse transcribed using iScript Reverse Transcription SuperMix (#1708840, Bio-Rad), according to the manufacturer’s protocol and as described [44]. RT-qPCR was performed using a Bio-Rad CFX96 Touch machine using SSoAdvanced Universal SYBR Green (#1725275, Bio-Rad) for visualization and quantification according to the manufacturer’s instructions. Primer sequences were: *Brca1*, forward: 5′-GCAGATGGGCTGACAGTAAA-3′, reverse: 5′-GCTTTCTACCACAGAGGGAATC-3′, TBP, forward: 5′-AGTGCCCAGCATCACTGTTT-3′, reverse: 5′-GGTCCATGACTCTCACTTTCTT-3′. Relative expression levels were calculated from the average threshold cycle number using the delta-delta Ct method.

### Fluorescence microscopy and image analysis

Cells were imaged with the Nikon A1R confocal laser microscope (Nikon Corporation) with the 100X Plan-Apo/1.4 NA oil lens. N-TASQ was imaged with the 488-nm laser, and the DAPI dye was imaged with the 405-nm laser. N-TASQ and γH2A.X fluorescence was analyzed by the puncta index, which is the standard deviation of the intensities measured among pixels within the neuronal nuclei. Low puncta index represents diffuse localization, whereas a high puncta index represents punctate localization.

### Immunocytochemistry and N-TASQ staining

Immunocytochemistry of primary cortical neurons, astrocytes, and microglial cells was as described [37, 67]. Briefly, cultured primary cortical neurons, astrocytes, and microglial cells grown on glass coverslips were fixed with 4% paraformaldehyde, permeabilized with a 0.01% Triton X-100/PBS solution, and blocked with a 5% bovine serum in PBS solution. In some experiments, cells were treated with a vehicle or 2 μM PDS overnight before fixation. Cells were then stained with primary antibodies (against MAP2c, GFAP, Iba-1 or γH2A.X) and with the G4-selective fluorophore N-TASQ overnight at 4° C in the dark. Cells were incubated with secondary antibodies, stained with Hoechst dye, and imaged with the Nikon A1R confocal laser microscope.

### Preparation of the oligonucleotides for CD and UV-melting experiments

The lyophilized DNA strands purchased from Eurogentec (Seraing, Belgium) were diluted at 500 μM in deionized water (18.2 MΩ.cm resistivity). The actual concentration of each solubilized sample was determined through a dilution to 5 μM theoretical concentration *via* a UV spectral analysis at 260 nm (after 5 min at 90° C) with the following molar extinction coefficient (ε) values: 251300 (hBrca1P), 243600 (hBrca1P^-^), 226300 (hBrca1Gb_1_), 218400 (hBrca1Gb_2_), 248600 (hBrca1Gb^-^), 274400 (mBrca1P), 247100 (mBrca1P^-^), 336200 (mBrca1Gb_1_), 289100 (mBrca1Gb_2_), 316900 (rBrca1P), 287000 (rBrca1P^-^), 216100 (rBrca1Gb_1_), and 250800 l.mol^-1^.cm^-1^ (rBrca1Gb_2_). For experiments in K^+^ conditions, DNA samples were prepared by mixing 10 μL of the constitutive strand (500 μM) with 10 μL of a lithium cacodylate buffer solution (100 mM, pH 7.2), plus 10 μL of a KCl/LiCl solution (100 mM/900 mM) and 70 μL of deionized water. For experiments in Na^+^ conditions, DNA samples were prepared by mixing 10 μL of the constitutive strand (500 μM) with 10 μL of a lithium cacodylate buffer solution (100 mM, pH 7.2), plus 10 μL of a NaCl (1 M) and 70 μL of deionized water. The G4 structures were folded by heating the solutions at 90° C for 5 min and then cooling them on ice (for 2 h) before storing them overnight at 4° C.

### CD and TDS experiments

CD and UV-Vis spectra were recorded on the JASCO J-815 spectropolarimeter in a 10-mm path-length quartz semi-micro cuvette (Starna). CD and UV-Vis spectra of 3 μM of DNA sample (Eurogentec) were recorded over 220–350 nm at 25 and 90° C (bandwidth = 1 nm, 1 nm data pitch, 1 s response, scan speed = 200 nm.mn^-1^, averaged over 4 scans) in 100 μL (final volume). Experiments in K^+^-conditions were performed in Caco.K10 buffer (10 mM lithium cacodylate buffer (pH 7.2) plus 10 mM KCl and 90 mM LiCl), and experiments in Na^+^ conditions contained Caco.Na100 buffer (10 mM lithium cacodylate buffer (pH 7.2) plus 100 mM NaCl). Final data were treated with OriginPro^®^9.1 (OriginLab Corp.). TDS signatures were calculated by subtracting the spectra collected at 25° C from those collected at 90° C and then zeroed at 350 nm. CD spectra were recorded on the JASCO J-815 spectropolarimeter in a 10-mm path-length quartz semi-micro cuvette (Starna). Spectra of 3 μM of DNA sample were recorded from 25–90° C at 264 and 320 nm (sampling = 2° C, ramp rate = 2° C.mn^-1^). Samples were prepared in 100 μL (final volume) of Caco.K10 buffer (10 mM lithium cacodylate buffer (pH 7.2) plus 10 mM KCl and 90 mM LiCl) with or without PDS (15 μM, 5 mol. equiv.). Final data from triplicates were treated with OriginPro^®^9.1 (OriginLab Corp.), subtracting the spectra collected at 320 nm from the spectra collected at 264 nm and averaging the triplicates.

## Supplementary Material

Supplementary Figures
