# Supplementary material for: Differential responses of neurons, astrocytes, and microglia to G-quadruplex stabilization
Source: Aging (Albany NY). 2021 Jun 19;13(12):15917–41. doi: 10.18632/aging.203222 (PMC8266374; doi:10.18632/aging.203222)
Supplement: Supplementary Figures [file aging-13-203222-s001.pdf]

SUPPLEMENTARY FIGURES

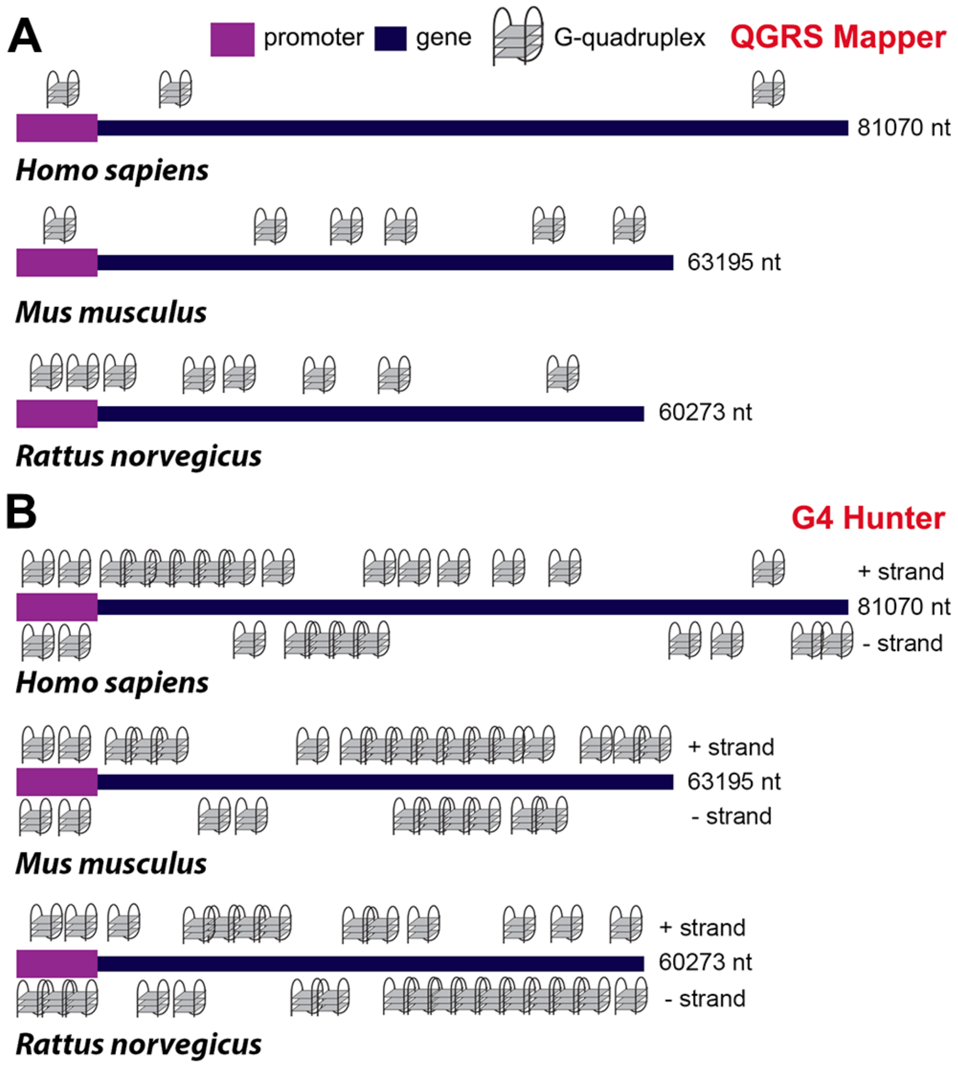

Supplementary Figure 1. PQFS in the gene and the promoter sequence of the *Brca1* in *Homo sapiens*, *Mus musculus*, and *Rattus norvegicus*. (**A**) The numbers of PQFS in *Brca1* and its promoter in *H. sapiens*, *M. musculus*, and *R. norvegicus* were analyzed using the QGRS mapper (http://bioinformatics.ramapo.edu/QGRS/index.php). 5000 nucleotides upstream of the gene was considered to be the promoter. NCBI Entrez Gene ID of *Brca1* in *H. sapiens* is 672; NCBI Entrez Gene ID of *Brca1* in *M. musculus* is 12189, and NCBI Entrez Gene ID of *Brca1* in *R. norvegicus* is 497672. (**B**) The numbers of PQFS in *Brca1* and its promoter in *H. sapiens* (672), *M. musculus* (12189), and *R. norvegicus* (497672) were analyzed using the G4 Hunter (https://bioinformatics.cruk.cam.ac.uk/G4Hunter/).

A

Name	Strand	Species	Location	Sequence	GH score	Line
**hBrca1P**	+	Human	Promoter	GGGCGGAAAGAGTGGGGTAATGGG	1.62	
**hBrca1P$^-$**	-	Human	Promoter	GGAGGGAACTTGAAGGGGTTTGGG	1.58	
**hBrca1Gb$_1$**	+	Human	Gene body	GGGGTAGGGGTGGATATGGGTG	2.09	
**hBrca1Gb$_2$**	+	Human	Gene body	GGACGGGGGACAGGCTGTGGGG	1.91	
**hBrca1Gb$^-$**	-	Human	Gene body	GGGATGGATCAGTGGGGGAAGTGG	1.58	
**mBrca1P**	+	Mouse	Promoter	GAGGTGGGCTGTGGGGAGGGTAAACGG	1.56	
**mBrca1P$^-$**	-	Mouse	Promoter	GGGGAAGGCCTGGGGAGAGGGATG	1.79	
**mBrca1Gb$_1$**	+	Mouse	Gene body	GAATCTGTGGGGTGGGGTGGAGTGGGGTGACTGG	1.71	
**mBrca1Gb$_2$**	+	Mouse	Gene body	GACTGGGGATGGAGGGTTCTGGAGGGTGG	1.55	
**rBrca1P**	+	Rat	Promoter	GGGCTCTGCCTTGGGGGGGGGGGCAGGAAGGG	1.94	
**rBrca1P$^-$**	-	Rat	Promoter	AAGGGGAGGGGGAGGGGTTAGGGGGAT	2.67	
**rBrca1Gb$_1$**	+	Rat	Gene body	GGGGACGGGGAGGGGCAAGTG	2.29	
**rBrca1Gb$_2$**	+	Rat	Gene body	GGGTGTTGGGGTCAGGCGAGGAGGG	1.68	

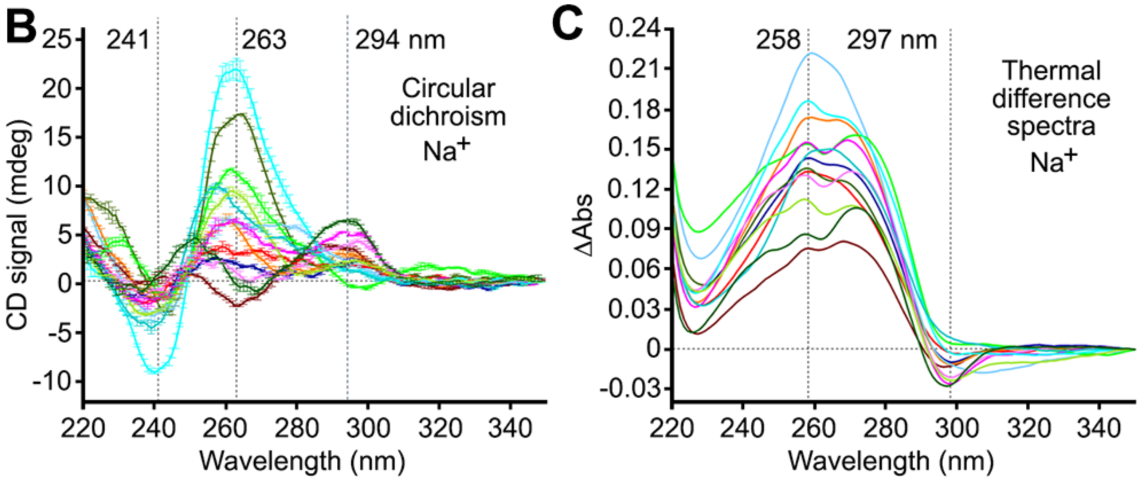

Supplementary Figure 2. (**A**) Sequences of putative G4-DNA-forming sequences from the human, mouse, and rat *Brca1* and their promoters, along with their G4Hunter (GH) scores. (**B**, **C**) CD and TDS signatures of these G4-forming sequences (3 µM) in Caco.Na100 buffer (10 mM lithium cacodylate buffer (pH 7.2) plus 100 mM NaCl).

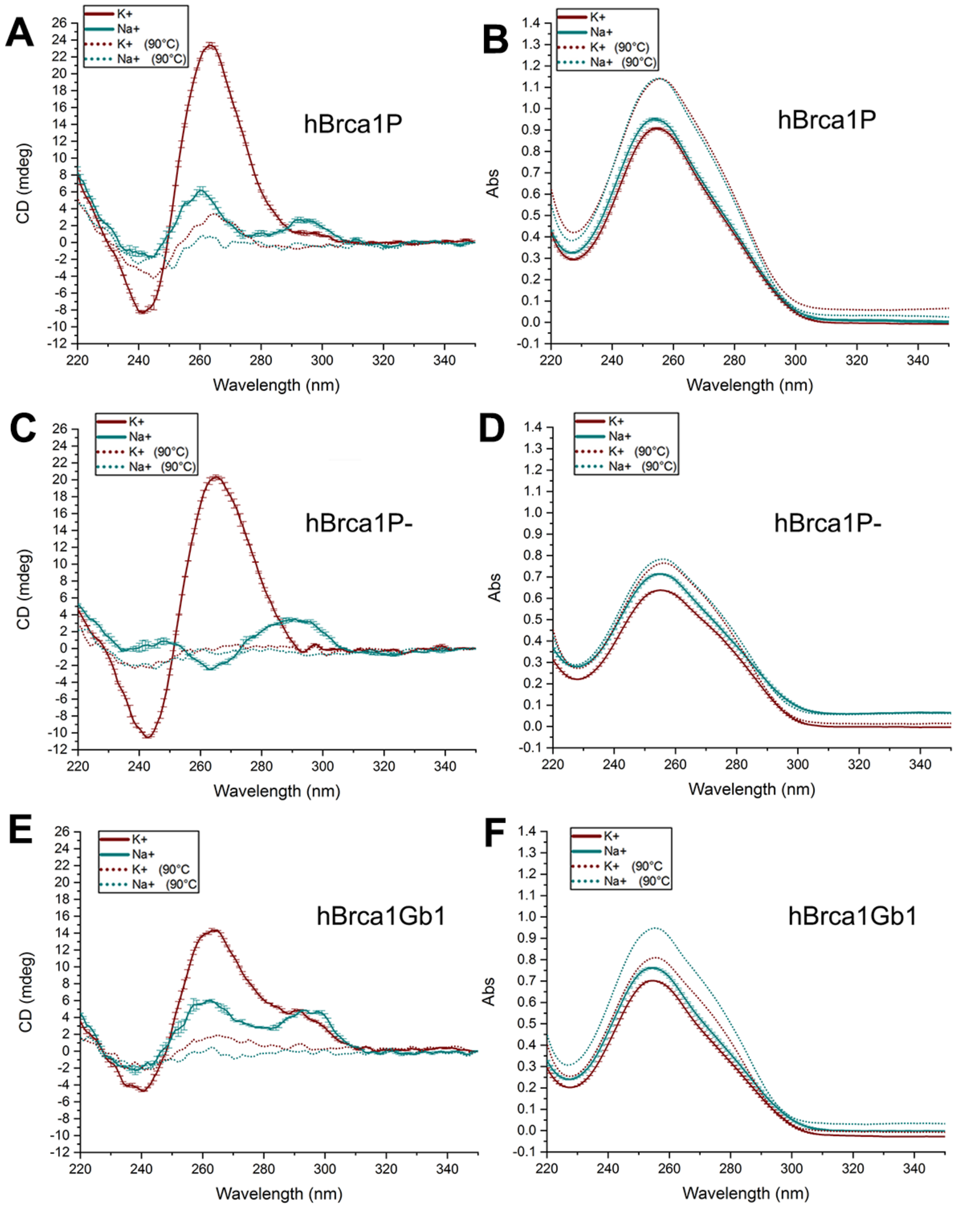

Supplementary Figure 3. (**A**, **B**) CD and UV-Vis signatures hBrca1P (3 μM) in CacoK10 or Caco.Na100 buffer, at 25 and 90° C. (**C**, **D**) CD and UV-Vis signatures hBrca1P^{-} (3 μM) in CacoK10 or Caco.Na100 buffer, at 25 and 90° C. (**E**, **F**) CD and UV-Vis signatures hBrca1Gb1 (3 μM) in CacoK10 or Caco.Na100 buffer, at 25 and 90° C.

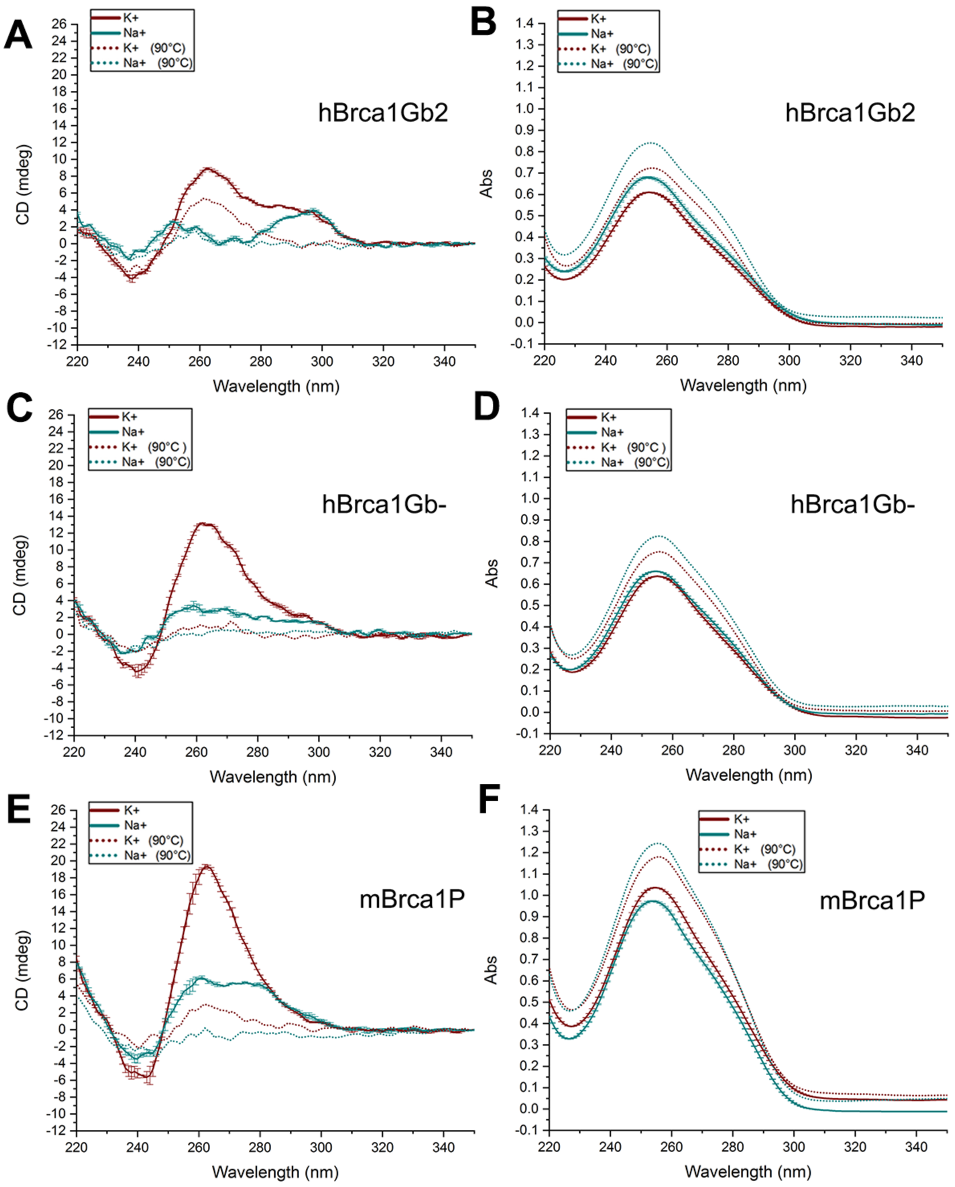

Supplementary Figure 4. (**A**, **B**) CD and UV-Vis signatures hBrca1Gb2 (3 µM) in CacoK10 or Caco.Na100 buffer, at 25 and 90° C. (**C**, **D**) CD and UV-Vis signatures hBrca1Gb⁻ (3 µM) in CacoK10 or Caco.Na100 buffer, at 25 and 90° C. (**E**, **F**) CD and UV-Vis signatures mBrca1P (3 µM) in CacoK10 or Caco.Na100 buffer, at 25 and 90° C.

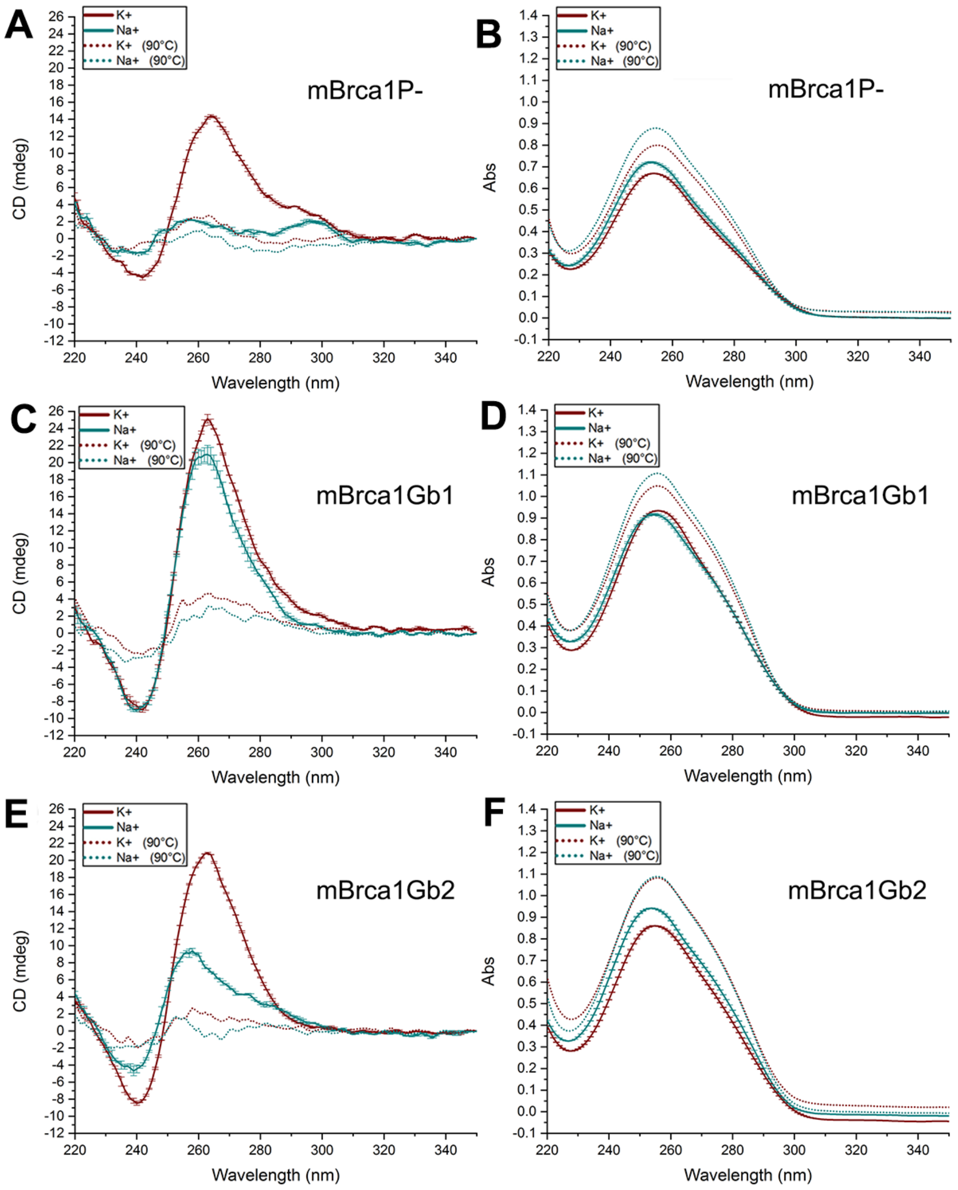

Supplementary Figure 5. (**A**, **B**) CD and UV-Vis signatures mBrca1P^{-} (3 µM) in CacoK10 or Caco.Na100 buffer, at 25 and 90° C. (**C**, **D**) CD and UV-Vis signatures mBrca1Gb1 (3 µM) in CacoK10 or Caco.Na100 buffer, at 25 and 90° C. (**E**, **F**) 6. CD and UV-Vis signatures mBrcaGb2 (3 µM) in CacoK10 or Caco.Na100 buffer, at 25 and 90° C.

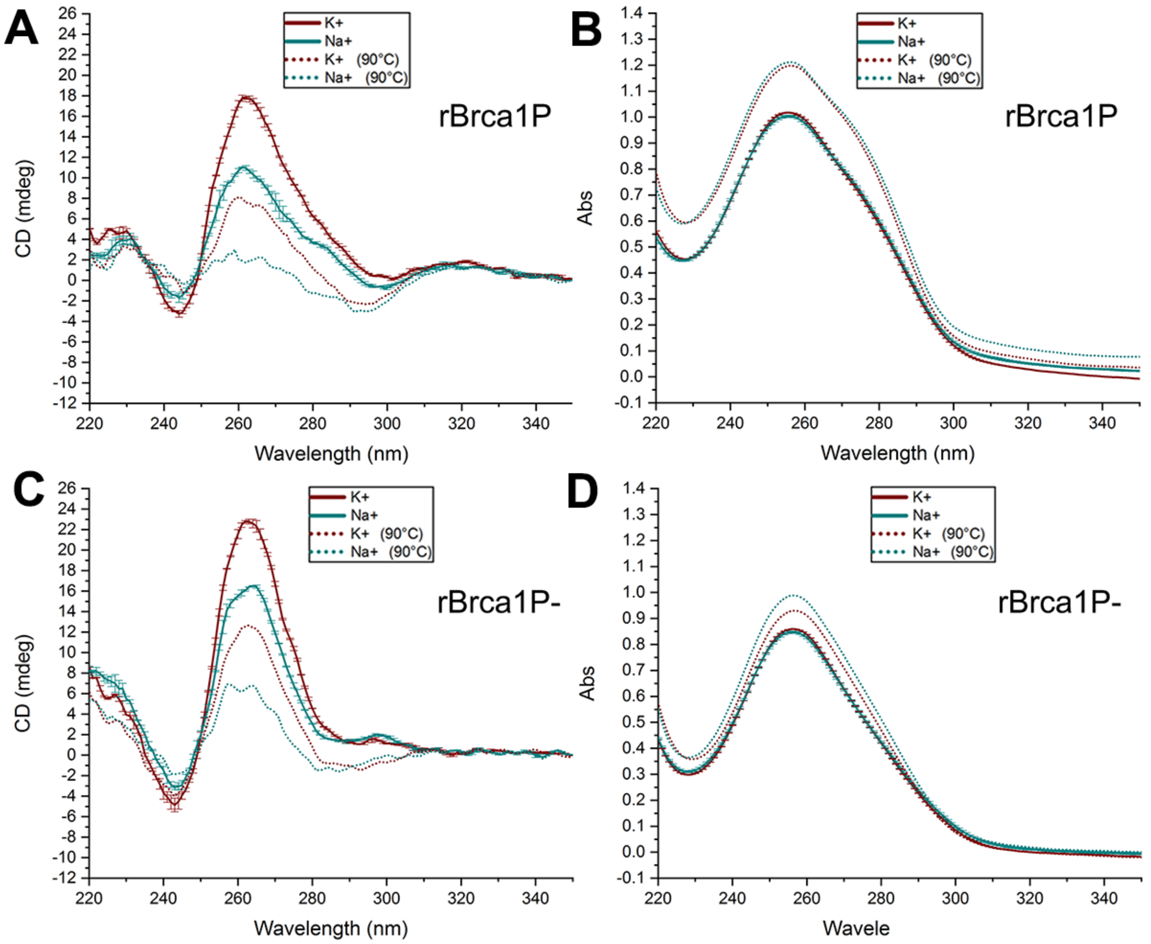

Supplementary Figure 6. (**A**, **B**) CD and UV-Vis signatures rBrca1P (3 µM) in CacoK10 or Caco.Na100 buffer, at 25 and 90° C. (**C**, **D**) CD and UV-Vis signatures rBrca1P^{-} (3 µM) in CacoK10 or Caco.Na100 buffer, at 25 and 90° C.

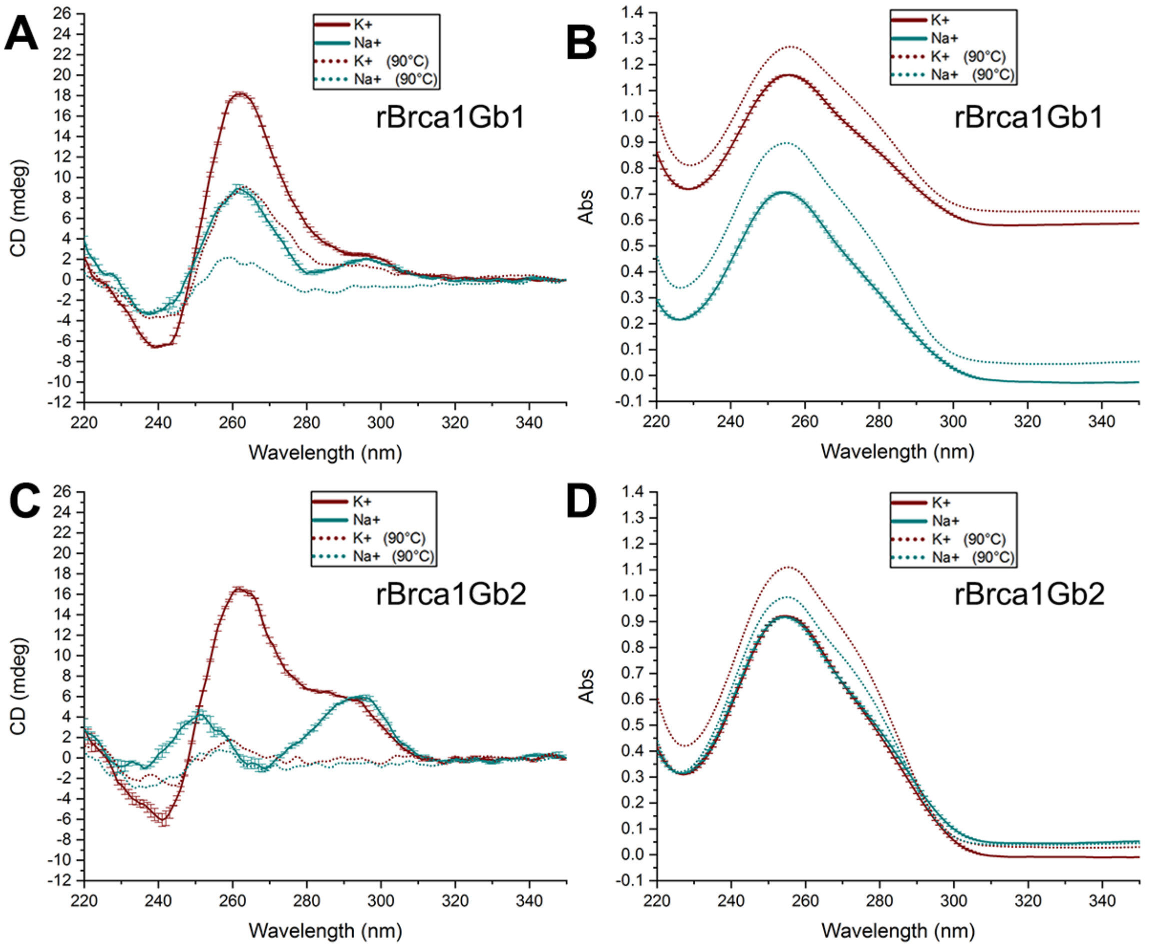

Supplementary Figure 7. (**A**, **B**) CD and UV-Vis signatures rBrca1Gb1 (3 µM) in CacoK10 or Caco.Na100 buffer, at 25 and 90° C. (**C**, **D**) CD and UV-Vis signatures rBrca1Gb2 (3 µM) in CacoK10 or Caco.Na100 buffer, at 25 and 90° C.

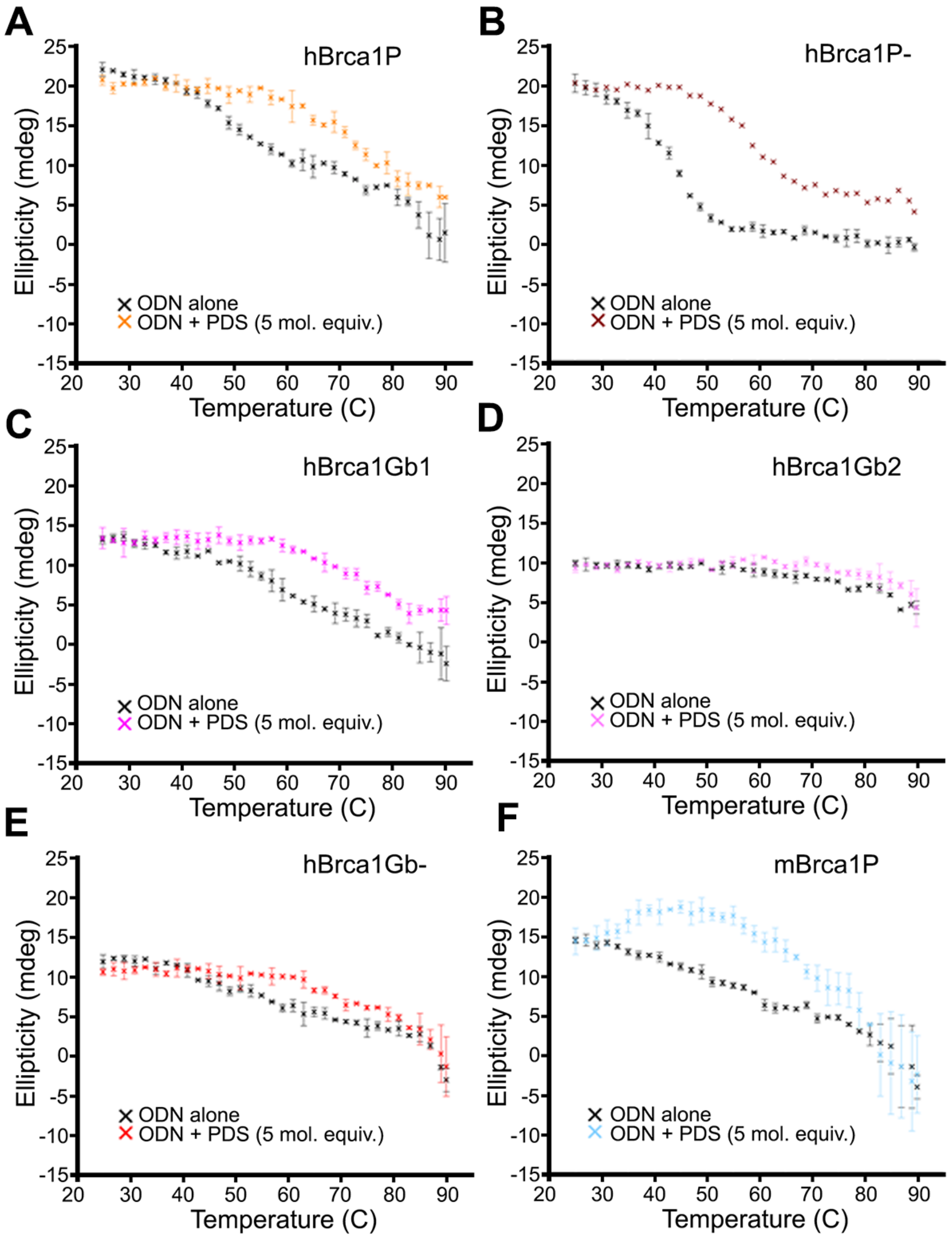

Supplementary Figure 8. (**A**) CD-melting experiments performed with hBrca1P (3 µM) in cacoK10 with or without PDS (15 µM) from 25 to 90° C. (**B**) CD-melting experiments performed with hBrca1P- (3 µM) in cacoK10 with or without PDS (15 µM) from 25 to 90° C. (**C**) CD-melting experiments performed with hBrca1Gb1 (3 µM) in cacoK10 with or without PDS (15 µM) from 25 to 90° C. (**D**) CD-melting experiments performed with hBrca1Gb2 (3 µM) in cacoK10 with or without PDS (15 µM) from 25 to 90° C. (**E**) CD-melting experiments performed with hBrca1Gb1- (3 µM) in cacoK10 with or without PDS (15 µM) from 25 to 90° C. (**F**) CD-melting experiments performed with mBrca1P (3 µM) in cacoK10 with or without PDS (15 µM) from 25 to 90° C.

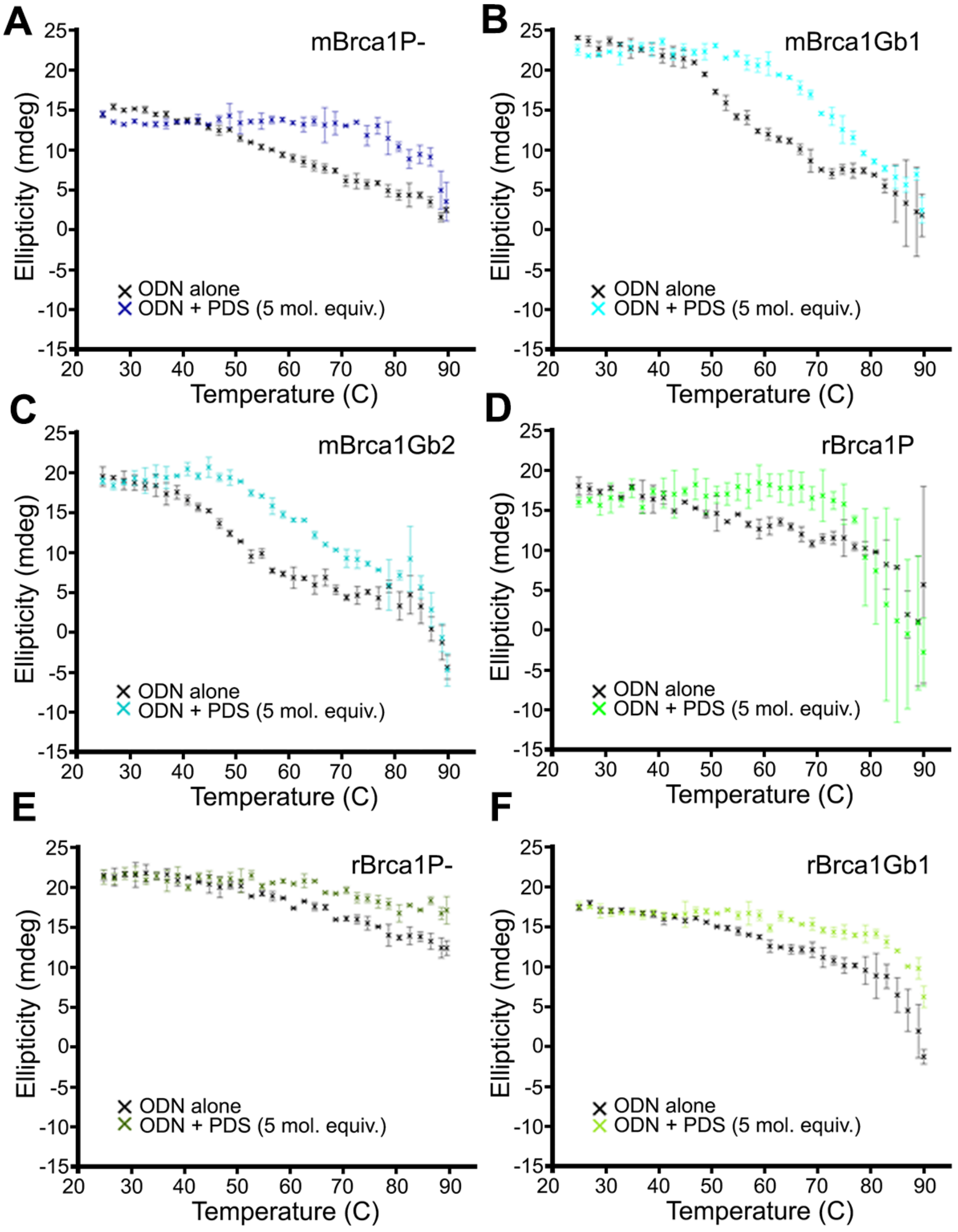

Supplementary Figure 9. (**A**) CD-melting experiments performed with mBrca1P$^-$ (3 µM) in cacoK10 with or without PDS (15 µM) from 25 to 90° C. (**B**) CD-melting experiments performed with mBrca1Gb1 (3 µM) in cacoK10 with or without PDS (15 µM) from 25 to 90° C. (**C**) CD-melting experiments performed with mBrca1Gb2 (3 µM) in cacoK10 with or without PDS (15 µM) from 25 to 90° C. (**D**) CD-melting experiments performed with rBrca1P (3 µM) in cacoK10 with or without PDS (15 µM) from 25 to 90° C. (**E**) CD-melting experiments performed with rBrca1P$^-$ (3 µM) in cacoK10 with or without PDS (15 µM) from 25 to 90° C. (**F**) CD-melting experiments performed with rBrca1Gb1 (3 µM) in cacoK10 with or without PDS (15 µM) from 25 to 90° C.

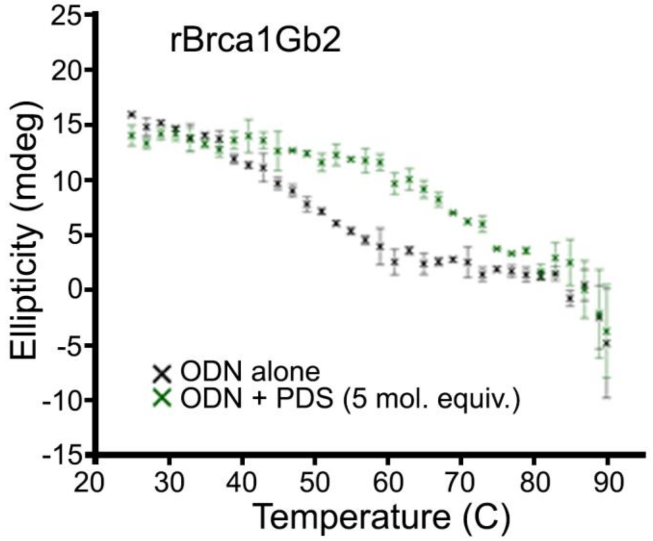

Supplementary Figure 10. CD-melting experiments performed with rBrca1Gb2 (3 µM) in cacoK10 with or without PDS (15 µM) from 25 to 90° C.
